# Bis[2-(1,3-benzo­thia­zol-2-yl)phenyl-κ^2^ *C* ^1^,*N*][1,3-bis­(4-bromo­phen­yl)propane-1,3-dionato-κ^2^ *O*,*O*′]iridium(III)

**DOI:** 10.1107/S1600536813018394

**Published:** 2013-07-10

**Authors:** Young-Inn Kim, Seong-Jae Yun, Sung Kwon Kang

**Affiliations:** a Department of Chemistry Education and Interdisciplinary Program of Advanced Information and Display Materials, Pusan National University, Busan 609-735, Republic of Korea; b Department of Chemistry, Chungnam National University, Daejeon 305-764, Republic of Korea

## Abstract

The title complex, [Ir(C_15_H_9_Br_2_O_2_)(C_13_H_8_NS)_2_], lies about a crystallographic twofold rotation axis passing through the Ir^III^ atom and the central C atom of the bis­(bromo­phen­yl)propane-1,3-dionate ligand. The Ir^III^ atom adopts a distorted octa­hedral geometry coordinated by two N atoms in the axial positions, and two C and two O atoms in the equatorial plane. The dihedral angle between the two thia­zole ring systems in the complex is 77.45 (10)°.

## Related literature

For luminescent Ir complexes, see: Ulbricht *et al.* (2009 ▶); Liu *et al.* (2008 ▶); Hwang *et al.* (2005 ▶); Tsuboyama *et al.* (2003 ▶); Bera *et al.* (2007 ▶). For phospho­rescent Ir complexes, see: Xu *et al.* (2009 ▶); Sengottuvelan *et al.* (2011 ▶, 2013 ▶).
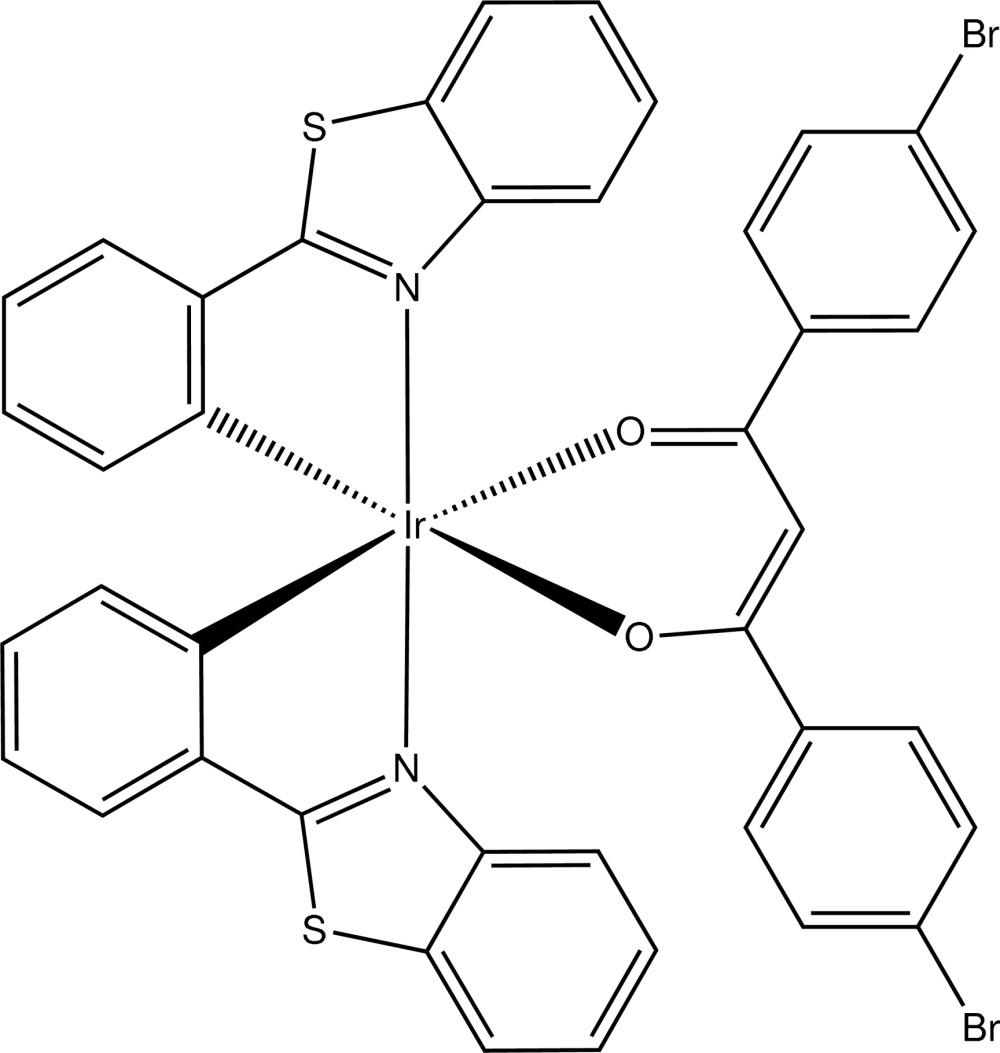


## Experimental

### 

#### Crystal data

[Ir(C_15_H_9_Br_2_O_2_)(C_13_H_8_NS)_2_]
*M* *_r_* = 993.77
Monoclinic, 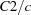

*a* = 15.888 (4) Å
*b* = 12.689 (3) Å
*c* = 17.143 (5) Å
β = 100.28 (5)°
*V* = 3400.8 (16) Å^3^
*Z* = 4
Mo *K*α radiation
μ = 6.44 mm^−1^
*T* = 203 K
0.35 × 0.29 × 0.16 mm

#### Data collection

Bruker SMART CCD area-detector diffractometer
Absorption correction: multi-scan (*SADABS*; Bruker, 2002 ▶) *T*_min_ = 0.132, *T*_max_ = 0.365
15842 measured reflections
4079 independent reflections
3623 reflections with *I* > 2σ(*I*)
*R*_int_ = 0.059

#### Refinement

*R*[*F* ^2^ > 2σ(*F* ^2^)] = 0.030
*wR*(*F* ^2^) = 0.076
*S* = 1.06
4079 reflections
227 parameters
H-atom parameters constrained
Δρ_max_ = 1.55 e Å^−3^
Δρ_min_ = −1.26 e Å^−3^

### 

Data collection: *SMART* (Bruker, 2002 ▶); cell refinement: *SAINT* (Bruker, 2002 ▶); data reduction: *SAINT*; program(s) used to solve structure: *SHELXS2013* (Sheldrick, 2013 ▶); program(s) used to refine structure: *SHELXL2013* (Sheldrick, 2013 ▶); molecular graphics: *ORTEP-3 for Windows* (Farrugia, 2012 ▶); software used to prepare material for publication: *WinGX* (Farrugia, 2012 ▶).

## Supplementary Material

Crystal structure: contains datablock(s) global, I. DOI: 10.1107/S1600536813018394/is5287sup1.cif

Structure factors: contains datablock(s) I. DOI: 10.1107/S1600536813018394/is5287Isup2.hkl

Additional supplementary materials:  crystallographic information; 3D view; checkCIF report

## Figures and Tables

**Table 1:** Selected bond lengths (Å)

Ir1—C18	1.996 (4)
Ir1—N12	2.060 (3)
Ir1—O2	2.155 (3)

**Fig. 1.**
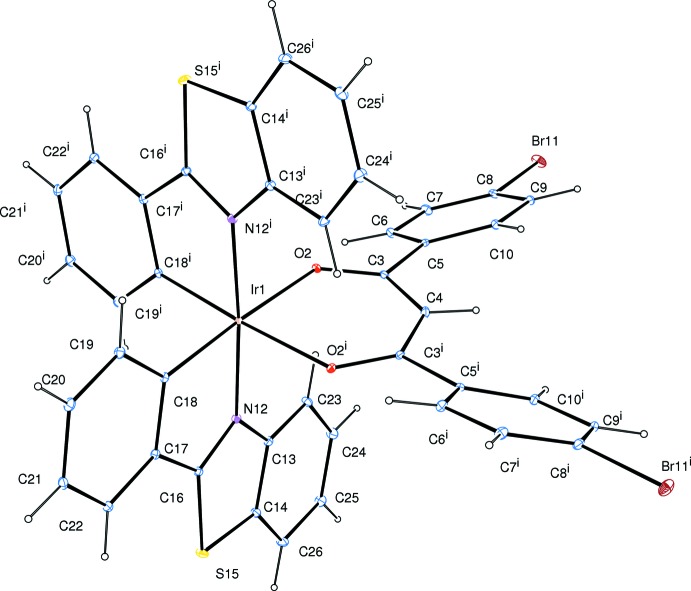
Molecular structure of the title compound, showing the atom-numbering scheme and 30% probability ellipsoids. [Symmetry code: (i) -x, y, -z + 1/2.]

## supplementary crystallographic information

### Comment

Electrophosphorescent materials based on iridium(III) have been developed in order to apply for organic light-emitting diodes (OLEDs) (Ulbricht *et al.*, 2009) because iridium(III) complexes possess relatively short excited state lifetimes, high quantum efficiencies (Liu *et al.*, 2008; Hwang *et al.*, 2005; Tsuboyama *et al.*, 2003) and remarkable color tuning from red to blue by a modification of the ligand structures (Bera *et al.*, 2007). Recently, we reported red phosphorescent iridium complexes (Sengottuvelan, Yun, Kim *et al.*, 2013; Sengottuvelan *et al.*, 2011) for an application for OLEDs. Herein, an orange-red emissive complex, a new heteroleptic cyclometalated iridium(III) complex containing two 2-phenylbenzothiazole as main ligands and 1,3-bis(*p*-bromophenyl)-1,3-propanedione as an ancillary ligand, is prepared and its crystal structure is reported. The title complex emitted at 617 (595 s h.) nm in dichloromethane at room temperature.

In the title compound (Fig. 1), the Ir^III^ atom lies on a twofold axis and is coordinated by two C atoms, two N atoms, and two O atoms of three bidentate ligands in a distorted octahedral geometry. The angles around Ir atoms are in the range of 79.67 (14)–97.27 (14)°. The Ir—C bond distances of 1.996 (4) Å are shorter than the Ir—N distances of 2.060 (3) Å due to the stronger *trans* influence of the phenyl ring compared to the 5-membered thiazole ring (Table 1). The bidentate 1,3-benzothiazol-2-ylphenyl ligand (N12–C26) is almost planar, with an r.m.s. deviation of 0.051 Å from the corresponding least-squares plane defined by the fifteen constituent atoms.

### Experimental

2-Phenylbenzothiazole (pbt) was purchased from Sigma-Aldrich Chemicals.

Synthesis of 1,3-bis(*p*-bromophenyl)-1,3-propanedione (dbacac): A mixture of a sodium hydride in oil dispersion (60%) and ethyl 4-bromobenzoate in 20 ml of dry THF was heated to 60 °C. 4-Bromoacetophenone in 8 ml in dry THF was added dropwise to the mixture. After the reaction temperature was held at 60 °C for 1 day, the mixture was poured into water and then neutralized with hydrochloric acid. The resulting precipitate was recrystallized from dichloromethane and hexane to give pale ivory powders.

Synthesis of title complex: The reaction of IrCl_3_ 3H_2_O with pbt in a 3:1 mixture of 2-ethoxyethanol and water at 135 °C gave cyclometallated iridium(III) µ-chloro-bridged dimer, [(pbt)_2_Ir(µ-Cl)]_2_. The prepared iridium(III) dimer complex, sodium carbonate and dbacac were dissolved in 2-ethoxyethanol. The mixture was heated at 125 °C for 8 h. The mixture was extracted with dichloromethane and dried over anhydrous magnesium sulfate. The crude product was flash chromatographed on silica gel using dichloromethane/methanol as an eluent. The red crystals were obtained from hexane/chloroform solution by slow evaporation at room temperature.

### Refinement

All H atoms were positioned geometrically and refined using a riding model, with C—H = 0.93 Å and with *U*_iso_(H) = 1.2*U*_eq_(C). The maximum and minimum residual electron density peaks were located at 0.84 and 0.81 Å, respectively, from atom Ir1.

### Crystal data

**Table:**

[Ir(C_15_H_9_Br_2_O_2_)(C_13_H_8_NS)_2_]	*F*(000) = 1920
*M**_r_* = 993.77	*D*_x_ = 1.941 Mg m^−^^3^
Monoclinic, *C*2/*c*	Mo *K*α radiation, λ = 0.71073 Å
Hall symbol: -C 2yc	Cell parameters from 6529 reflections
*a* = 15.888 (4) Å	θ = 2.3–28.2°
*b* = 12.689 (3) Å	µ = 6.44 mm^−^^1^
*c* = 17.143 (5) Å	*T* = 203 K
β = 100.28 (5)°	Block, red
*V* = 3400.8 (16) Å^3^	0.35 × 0.29 × 0.16 mm
*Z* = 4	

### Data collection

**Table:**

Bruker SMART CCD area-detector diffractometer	3623 reflections with *I* > 2σ(*I*)
Radiation source: fine-focus sealed tube	*R*_int_ = 0.059
φ and ω scans	θ_max_ = 28.3°, θ_min_ = 2.1°
Absorption correction: multi-scan (*SADABS*; Bruker, 2002)	*h* = −9→21
*T*_min_ = 0.132, *T*_max_ = 0.365	*k* = −16→8
15842 measured reflections	*l* = −21→21
4079 independent reflections	

### Refinement

**Table:**

Refinement on *F*^2^	0 restraints
Least-squares matrix: full	Hydrogen site location: inferred from neighbouring sites
*R*[*F*^2^ > 2σ(*F*^2^)] = 0.030	H-atom parameters constrained
*wR*(*F*^2^) = 0.076	*w* = 1/[σ^2^(*F*_o_^2^) + (0.0363*P*)^2^ + 6.8133*P*] where *P* = (*F*_o_^2^ + 2*F*_c_^2^)/3
*S* = 1.06	(Δ/σ)_max_ = 0.001
4079 reflections	Δρ_max_ = 1.55 e Å^−^^3^
227 parameters	Δρ_min_ = −1.26 e Å^−^^3^

### Special details

**Table:**

Geometry. All e.s.d.'s (except the e.s.d. in the dihedral angle between two l.s. planes) are estimated using the full covariance matrix. The cell e.s.d.'s are taken into account individually in the estimation of e.s.d.'s in distances, angles and torsion angles; correlations between e.s.d.'s in cell parameters are only used when they are defined by crystal symmetry. An approximate (isotropic) treatment of cell e.s.d.'s is used for estimating e.s.d.'s involving l.s. planes.

### Fractional atomic coordinates and isotropic or equivalent isotropic displacement parameters (Å^2^)

**Table:**

	*x*	*y*	*z*	*U*_iso_*/*U*_eq_	
Ir1	0	0.38790 (2)	0.25	0.01610 (7)	
O2	0.02867 (16)	0.2664 (2)	0.33857 (15)	0.0201 (5)	
C3	0.0262 (2)	0.1685 (3)	0.3239 (2)	0.0198 (7)	
C4	0	0.1208 (4)	0.25	0.0307 (13)	
H4	0	0.0475	0.25	0.037*	
C5	0.0587 (2)	0.0971 (3)	0.3927 (2)	0.0206 (7)	
C6	0.1155 (3)	0.1359 (3)	0.4576 (2)	0.0270 (8)	
H6	0.1298	0.207	0.4595	0.032*	
C7	0.1512 (3)	0.0701 (4)	0.5198 (3)	0.0311 (9)	
H7	0.1909	0.0961	0.562	0.037*	
C8	0.1264 (3)	−0.0348 (3)	0.5177 (3)	0.0290 (9)	
C9	0.0688 (3)	−0.0745 (3)	0.4554 (2)	0.0265 (8)	
H9	0.0528	−0.145	0.4547	0.032*	
C10	0.0349 (3)	−0.0081 (3)	0.3935 (2)	0.0244 (8)	
H10	−0.0047	−0.0346	0.3516	0.029*	
Br11	0.17062 (3)	−0.12509 (4)	0.60339 (3)	0.04469 (14)	
N12	0.11887 (19)	0.3940 (2)	0.21803 (19)	0.0202 (6)	
C13	0.1968 (2)	0.3442 (3)	0.2459 (2)	0.0207 (7)	
C14	0.2596 (2)	0.3667 (3)	0.1998 (2)	0.0244 (8)	
S15	0.21858 (6)	0.45204 (9)	0.12315 (6)	0.0295 (2)	
C16	0.1222 (2)	0.4541 (3)	0.1558 (2)	0.0199 (7)	
C17	0.0476 (2)	0.5149 (3)	0.1241 (2)	0.0196 (7)	
C18	−0.0208 (2)	0.4977 (3)	0.1654 (2)	0.0184 (7)	
C19	−0.0952 (2)	0.5581 (3)	0.1394 (2)	0.0261 (8)	
H19	−0.1418	0.5513	0.165	0.031*	
C20	−0.0997 (3)	0.6271 (3)	0.0762 (3)	0.0303 (9)	
H20	−0.1496	0.6655	0.0603	0.036*	
C21	−0.0318 (3)	0.6408 (3)	0.0359 (2)	0.0268 (9)	
H21	−0.0362	0.6875	−0.0064	0.032*	
C22	0.0422 (2)	0.5837 (3)	0.0601 (2)	0.0228 (8)	
H22	0.0883	0.5911	0.0337	0.027*	
C23	0.2167 (2)	0.2787 (3)	0.3110 (2)	0.0268 (8)	
H23	0.1763	0.2638	0.3425	0.032*	
C24	0.2977 (3)	0.2359 (4)	0.3284 (3)	0.0333 (10)	
H24	0.3115	0.1915	0.372	0.04*	
C25	0.3590 (3)	0.2575 (4)	0.2825 (3)	0.0329 (10)	
H25	0.413	0.2272	0.2954	0.039*	
C26	0.3408 (3)	0.3236 (4)	0.2180 (3)	0.0327 (9)	
H26	0.382	0.3388	0.1873	0.039*	

### Atomic displacement parameters (Å^2^)

**Table:**

	*U*^11^	*U*^22^	*U*^33^	*U*^12^	*U*^13^	*U*^23^
Ir1	0.01424 (10)	0.01535 (10)	0.01891 (12)	0	0.00346 (7)	0
O2	0.0234 (13)	0.0187 (13)	0.0190 (13)	−0.0013 (10)	0.0061 (10)	0.0028 (9)
C3	0.0209 (16)	0.0198 (18)	0.0201 (19)	−0.0006 (14)	0.0074 (14)	0.0040 (13)
C4	0.051 (4)	0.017 (3)	0.025 (3)	0	0.011 (3)	0
C5	0.0246 (18)	0.0197 (18)	0.0198 (19)	0.0025 (14)	0.0105 (14)	−0.0005 (13)
C6	0.0275 (19)	0.026 (2)	0.028 (2)	−0.0048 (16)	0.0062 (16)	0.0025 (15)
C7	0.0256 (19)	0.038 (2)	0.029 (2)	−0.0043 (18)	0.0026 (17)	0.0056 (17)
C8	0.0233 (18)	0.032 (2)	0.034 (2)	0.0048 (17)	0.0113 (17)	0.0138 (17)
C9	0.035 (2)	0.0183 (17)	0.029 (2)	0.0041 (16)	0.0139 (17)	0.0062 (15)
C10	0.0297 (19)	0.0208 (18)	0.024 (2)	−0.0005 (15)	0.0071 (16)	0.0023 (14)
Br11	0.0279 (2)	0.0540 (3)	0.0503 (3)	0.0050 (2)	0.0020 (2)	0.0296 (2)
N12	0.0169 (14)	0.0209 (15)	0.0228 (16)	0.0005 (12)	0.0034 (12)	−0.0015 (12)
C13	0.0143 (15)	0.0221 (18)	0.025 (2)	−0.0021 (14)	0.0016 (14)	−0.0042 (14)
C14	0.0207 (18)	0.025 (2)	0.028 (2)	0.0032 (15)	0.0063 (15)	0.0046 (14)
S15	0.0228 (5)	0.0371 (6)	0.0312 (6)	0.0040 (4)	0.0114 (4)	0.0114 (4)
C16	0.0198 (17)	0.0213 (17)	0.0185 (18)	−0.0025 (14)	0.0035 (14)	−0.0005 (13)
C17	0.0200 (17)	0.0193 (17)	0.0182 (18)	0.0002 (14)	0.0001 (14)	−0.0018 (13)
C18	0.0192 (16)	0.0154 (16)	0.0195 (19)	0.0010 (13)	0.0008 (14)	−0.0028 (12)
C19	0.0221 (18)	0.025 (2)	0.030 (2)	0.0033 (15)	0.0022 (16)	0.0000 (15)
C20	0.030 (2)	0.027 (2)	0.032 (2)	0.0094 (17)	0.0010 (17)	0.0011 (16)
C21	0.038 (2)	0.0191 (18)	0.022 (2)	−0.0004 (16)	0.0017 (17)	0.0013 (14)
C22	0.0251 (18)	0.0228 (18)	0.0200 (19)	−0.0028 (15)	0.0026 (15)	−0.0013 (14)
C23	0.0187 (17)	0.029 (2)	0.033 (2)	0.0008 (15)	0.0062 (16)	0.0096 (16)
C24	0.028 (2)	0.036 (2)	0.035 (2)	0.0082 (18)	0.0020 (18)	0.0116 (18)
C25	0.0216 (19)	0.035 (2)	0.041 (3)	0.0119 (17)	0.0036 (18)	0.0038 (18)
C26	0.0225 (19)	0.038 (2)	0.041 (3)	0.0040 (18)	0.0143 (17)	0.0054 (19)

### Geometric parameters (Å, º)

**Table:**

Ir1—C18^i^	1.996 (4)	C13—C23	1.382 (5)
Ir1—C18	1.996 (4)	C13—C14	1.407 (5)
Ir1—N12^i^	2.060 (3)	C14—C26	1.385 (5)
Ir1—N12	2.060 (3)	C14—S15	1.738 (4)
Ir1—O2^i^	2.155 (3)	S15—C16	1.722 (4)
Ir1—O2	2.155 (3)	C16—C17	1.436 (5)
O2—C3	1.266 (4)	C17—C22	1.394 (5)
C3—C4	1.399 (4)	C17—C18	1.415 (5)
C3—C5	1.505 (5)	C18—C19	1.413 (5)
C4—C3^i^	1.399 (4)	C19—C20	1.384 (6)
C4—H4	0.93	C19—H19	0.93
C5—C10	1.388 (5)	C20—C21	1.392 (6)
C5—C6	1.391 (6)	C20—H20	0.93
C6—C7	1.392 (6)	C21—C22	1.381 (6)
C6—H6	0.93	C21—H21	0.93
C7—C8	1.387 (6)	C22—H22	0.93
C7—H7	0.93	C23—C24	1.380 (5)
C8—C9	1.372 (6)	C23—H23	0.93
C8—Br11	1.896 (4)	C24—C25	1.384 (6)
C9—C10	1.386 (5)	C24—H24	0.93
C9—H9	0.93	C25—C26	1.377 (6)
C10—H10	0.93	C25—H25	0.93
N12—C16	1.320 (5)	C26—H26	0.93
N12—C13	1.396 (5)		
			
C18^i^—Ir1—C18	91.4 (2)	C13—N12—Ir1	133.7 (3)
C18^i^—Ir1—N12^i^	79.67 (14)	C23—C13—N12	127.6 (3)
C18—Ir1—N12^i^	97.27 (14)	C23—C13—C14	119.5 (3)
C18^i^—Ir1—N12	97.27 (14)	N12—C13—C14	113.0 (3)
C18—Ir1—N12	79.67 (14)	C26—C14—C13	121.3 (4)
N12^i^—Ir1—N12	175.67 (17)	C26—C14—S15	128.7 (3)
C18^i^—Ir1—O2^i^	176.72 (12)	C13—C14—S15	110.1 (3)
C18—Ir1—O2^i^	90.04 (12)	C16—S15—C14	89.93 (18)
N12^i^—Ir1—O2^i^	97.24 (11)	N12—C16—C17	117.8 (3)
N12—Ir1—O2^i^	85.88 (11)	N12—C16—S15	114.8 (3)
C18^i^—Ir1—O2	90.04 (12)	C17—C16—S15	127.3 (3)
C18—Ir1—O2	176.72 (12)	C22—C17—C18	123.1 (3)
N12^i^—Ir1—O2	85.88 (11)	C22—C17—C16	124.4 (4)
N12—Ir1—O2	97.24 (11)	C18—C17—C16	112.5 (3)
O2^i^—Ir1—O2	88.63 (14)	C19—C18—C17	115.6 (3)
C3—O2—Ir1	124.5 (2)	C19—C18—Ir1	129.0 (3)
O2—C3—C4	126.8 (4)	C17—C18—Ir1	115.4 (3)
O2—C3—C5	116.2 (3)	C20—C19—C18	120.9 (4)
C4—C3—C5	117.0 (4)	C20—C19—H19	119.5
C3^i^—C4—C3	128.7 (5)	C18—C19—H19	119.5
C3^i^—C4—H4	115.7	C19—C20—C21	122.1 (4)
C3—C4—H4	115.7	C19—C20—H20	119
C10—C5—C6	118.1 (4)	C21—C20—H20	119
C10—C5—C3	122.1 (3)	C22—C21—C20	118.6 (4)
C6—C5—C3	119.8 (3)	C22—C21—H21	120.7
C5—C6—C7	121.2 (4)	C20—C21—H21	120.7
C5—C6—H6	119.4	C21—C22—C17	119.6 (4)
C7—C6—H6	119.4	C21—C22—H22	120.2
C8—C7—C6	118.7 (4)	C17—C22—H22	120.2
C8—C7—H7	120.7	C24—C23—C13	118.8 (4)
C6—C7—H7	120.7	C24—C23—H23	120.6
C9—C8—C7	121.3 (4)	C13—C23—H23	120.6
C9—C8—Br11	119.0 (3)	C23—C24—C25	121.5 (4)
C7—C8—Br11	119.7 (3)	C23—C24—H24	119.3
C8—C9—C10	119.1 (4)	C25—C24—H24	119.3
C8—C9—H9	120.4	C26—C25—C24	120.6 (4)
C10—C9—H9	120.4	C26—C25—H25	119.7
C9—C10—C5	121.5 (4)	C24—C25—H25	119.7
C9—C10—H10	119.2	C25—C26—C14	118.3 (4)
C5—C10—H10	119.2	C25—C26—H26	120.8
C16—N12—C13	112.2 (3)	C14—C26—H26	120.8
C16—N12—Ir1	114.0 (2)		

Symmetry code: (i) −*x*, *y*, −*z*+1/2.
